# A tale of two time periods: ovarian cancer trends in Ontario

**DOI:** 10.3747/co.2007.106

**Published:** 2007-04

**Authors:** L. Elit, S.J. Bondy, Z. Chen, L. Paszat

**Affiliations:** * Department of Obstetrics and Gynecology, McMaster University, Hamilton, Ontario; † Department of Public Health Sciences, University of Toronto, Toronto, Ontario.; ‡ Institute for Clinical Evaluative Sciences, Toronto, Ontario.; § Department of Radiation Oncology and of Health Policy Management and Evaluation, University of Toronto, Toronto, Ontario

**Keywords:** Ovarian cancer, outcomes

## Abstract

We assessed population-based trends in incidence and survival rates for epithelial ovarian cancer in Ontario in two time periods. Our population-based study cohort included all women with epithelial ovarian cancer treated initially with abdominal surgery in Ontario for January 1996 through December 2001. Incident surgical cases were documented by hospital contact data and the Ontario Cancer Registry. Patient characteristics (age, for example) were obtained from electronic administrative data records. Regression analyses were used to assess the influence of time period on survival while controlling for age, comorbidity, and other factors associated with this outcome.

A total of 3825 women met the inclusion criteria. We found that the age-standardized incidence of ovarian cancer remained stable during 1996–2001. A shift to a younger age at diagnosis was found between the two time periods being compared. The univariate analysis revealed a clear difference in death rate, to which age at diagnosis, Charlson comorbidity score, and treatment period contributed. Earlier time period (*p* < 0.0001), advancing age (*p* < 0.0001), higher Charlson score (*p* < 0.0001), and lower income quartile score (*p* = 0.03) were significantly associated with poorer survival in the univariate analysis. Younger age, lower Charlson score, and more recent time period of diagnosis and treatment (*p* < 0.0001) were associated with improved survival in the proportional hazards model.

We conclude that age-standardized incidence and mortality rates for ovarian cancer in Ontario have remained stable. For women initially treated with surgery, advances in management have led to an improvement in survival.

## 1. INTRODUCTION

Ovarian cancer is the leading cause of gynecologic cancer death in women. Management involves a combination of surgery and adjuvant chemotherapy. Surgery is important for making the diagnosis, identifying prognostic factors, alleviating symptoms, and extending survival. Adjuvant chemotherapy usually involves a combination of platinum and taxanes^1^.

In 2005, we reviewed outcomes in women who had received chemotherapy for ovarian cancer during 1995–2002 2. We showed that provider discipline and provider’s volume of ovarian cancer chemotherapy cases did not affect patient survival. In that work, we noted a trend toward improved survival for the later period of study. In that paper, we suggested that advances in systemic therapy were likely responsible for the observed trend.

The objective of the present study was to analyze and compare trends in the incidence and survival rates for epithelial ovarian cancer among women who received surgery as initial treatment (with or without adjuvant chemotherapy) during 1996–1998 and 1999–2001. Factors that could influence survival rates were assessed.

## 2. PATIENTS AND METHODS

### 2.1 Data Sources and Elements

Our population-based cohort study included all women with newly diagnosed ovarian cancer treated initially with abdominal surgery in Ontario from January 1, 1996, to December 31, 2001. The Canadian Institute for Health Information (cihi) program gathers all hospital discharge data. The Ontario Cancer Registry (ocr) gathers all malignant histology information related to patients as provided by all Ontario hospital and outpatient laboratories. The cihi and ocr data were used to identify patients with ovarian cancer (International Classification of Disease code 10, diagnostic code 183).

The cihi records identified 5240 ovarian cancer patients in the study period. Of these, 1415 were excluded based on a prior ovarian cancer diagnosis between 1988 and December 31, 1995 (*n* = 18), age less than 18 years (*n* = 10), surgery completed out of province (*n* < 5), histology showing low malignant potential disease or other non-epithelial tumour types (*n* = 1372), or index ovarian cancer surgery occurring before January 1, 1996, or after December 31, 2001. The study database consisted of 3825 incident cases that were identified for 1996–2001 in the ocr by a process of case ascertainment consisting of deterministic linkage of records from the cancer centres, pathology reports from the Ontario hospitals, hospital discharge abstracts from cihi, and death certificates from the registrar general.

Information concerning mortality (death from any cause after the diagnosis with ovarian cancer) was obtained from the Registered Persons Database of the Ontario Health Insurance Program, the ocr, and the cihi. Survival follow-up was complete to March 2006, resulting in total follow-up times ranging from 1 day to 10.4 years. Dates of birth and diagnosis were obtained from the ocr and the cihi. Comorbidity was documented from cihi diagnosis codes using the Charlson–Deyo comorbidity score 3.

Ontario is divided into 14 local health integrated networks (lhins) that implement a regional approach to planning, integrating, and funding local health services. Regional residence of patients (by lhin) was examined as having a potential influence on survival, as were quintiles of household income by location of the patients’ residences, as assigned from Canadian census data matched to the postal code of each patient’s residence.

Analysis of data on survival time used the Kaplan–Meier estimation of survival functions and log-rank tests for unadjusted survival time comparisons. The Cox proportional hazards model was used to assess the significance of differences in mortality rates between time periods, adjusting for differences in other predictors of survival between the two time periods. Model diagnostics included assessment of assumptions of the Cox model.

## 3. RESULTS

In Ontario, the average number of patients receiving initial surgery for treatment of their epithelial ovarian cancer ranged from a low of 588 cases in calendar year 1997 to a high of 708 cases in 2001. The age-standardized incidence of epithelial ovarian cancer in Ontario was 14 per 100,000 woman–years in both 1996 4 and 2001 5. This incidence is slightly higher than that reported in Canada in 1996 4 (13 per 100,000) and in 2001 5 (12 per 100,000).

### 3.1 Deaths and Proportion Deceased

Table I presents numbers of patients and observed deaths in the period of follow-up by time period and patient characteristics. Across the two time periods, 2356 deaths were observed (61.6% of the cohort). Using chi-square analysis, the age distribution (*p* = 0.0002)—but not the Charlson comorbidity score (*p* = –0.86), income quintile (*p* = –0.45), or lhin (*p* = 0.38)—was observed to be different between the two time periods. The average number of ovarian cancer patients per region (lhin) ranged from 61 to 429 over the 6-year period (data by lhin not shown because of small numbers and confidentiality concerns).

Death rates were higher in the earlier time period (attributable to longer follow-up). The crude proportion of deaths was also higher among patients with more advanced age and higher Charlson comorbidity score at diagnosis (Table II). In Ontario, with its socialized medical care system, income quintile and lhin of residence were not associated with mortality.

### 3.2 Duration of Survival

Univariate analysis by the log-rank test or unadjusted Cox model for survival, using days to death, showed that the earlier time period, advancing age, higher Charlson score, and lower income quintile scores were each statistically significantly associated with a lower duration of survival (Tables III and IV). Where a patient lived in Ontario (lhin) did not affect duration of survival; however, the median duration of survival was lowest in the lhin with the lowest number of cases (that is, in a less densely populated region). The proportional hazards regression model showed that age, Charlson score, and time period for treatment all affected survival (*p* < 0.0001; Table V).

After consideration of differences in patient age and comorbidity between the two time periods, expected patient survival was improved in 1999–2001 as compared with 1996–1998. In the more recent period, patients were experiencing 90% of the mortality observed in the earlier time period per follow-up time unit.

## 4. DISCUSSION

Our analysis demonstrates that patient survival after epithelial ovarian cancer was significantly improved in 1999–2001 as compared to that observed in 1996–1998. Our analysis also confirms the importance to survival of comorbidity and age at diagnosis. We observed a shift toward a younger age at diagnosis between the two time periods, but that difference did not explain the improvement in survival.

In a socialized health care system, income strata and geographic region of residence did not affect the death rate. In the univariate analysis, duration of survival was associated with age, Charlson comorbidity score, income quintile, and treatment period. However, only treatment period, age, and Charlson score were important in the regression model. Despite an almost doubling of the age-standardized incidence of ovarian cancer, advances in management have led to an improvement in survival.

The age-standardized mortality rates in Ontario for epithelial ovarian cancer remained stable over the two time periods. The observed rates accord with those reported from other developed countries 6–9. The decline in the death rate in women who had surgery as the initial step in their management and the improved duration of survival in the later time period reflect either the earlier stage of diagnosis or improved treatment, or both.

Although screening for ovarian cancer is not advocated in Ontario, awareness is increasing among family doctors and patients about the importance of ultrasound and tumour markers such as cancer antigen 125 for women with symptoms suggestive of ovarian masses. Prophylactic surgery is also available for women with known *BRCA1, BRCA2,* or *MSH2* mutations. These manoeuvres provide the potential to identify the disease at an earlier stage. To determine if a stage shift is occurring, the stages of disease between the two time periods might have been compared. But although stage information is available for the earlier cohort, it is not available for the later cohort 10.

Although not observed directly in the present study, improved standards of care for ovarian cancer are likely to be the explanatory factor for the improved survival seen in the study cohort from the more recent period. During 1994–1995, cancer centres across Canada participated in a randomized trial of platinum with either cyclophosphamide or taxanes (National Cancer Institute of Canada trial OV10) 11. Results from that study were published in 2000. Subsequently, in September 2001, Cancer Care Ontario’s Program in Evidence-Based Care published a practice guideline making platinum and taxanes first-line therapy for advanced ovarian cancer 12. The use of platinum–taxane therapy was increasing in Ontario before adoption of the guideline. With the training of an increased number of gynecologic oncologists in Ontario from the 1990s onwards, a trend toward more aggressive surgical staging in early disease and tumour debulking for advanced disease may also have been occurring, together with a shift in referrals to gynecologic oncologists. Discipline of the primary surgeon involved in the operation has clearly been shown to affect survival.

The limitations of the present work include the lack of detailed information in the most recent cohort for disease-related variables (for example, stage) and for treatment (for example, chemotherapy agents used) 10. Thus, our findings of survival benefit based on management strategy are hypothesis-generating only. Age-standardized incidence and mortality rates for ovarian cancer in Ontario have remained stable. For women initially treated with surgery, advances in management are likely to have led to the observed improvement in survival.

## Figures and Tables

**TABLE I:** Patient characteristics, 1996–2001^a^

	*1996–1998 [*n *(%)]*	*1999–2001 [*n *(%)]*	*Overall [*n *(%)]*
Patients	1852	1973	3825
Age (years)			
18–49	409 (22.1)	488 (24.7)	897 (23.5)
50–59	396 (21.4)	508 (25.8)	904 (23.6)
60–69	486 (26.2)	457 (23.2)	943 (24.7)
70+	561 (30.3)	520 (26.4)	1081 (28.3)
cci			
0	577 (31.2)	680 (34.5)	1257 (32.9)
1–5	347 (18.7)	361 (18.3)	708 (18.5)
≥6	928 (50.1)	932 (47.2)	1860 (48.6)
Income quintile			
1	356 (19.9)	339 (17.6)	695 (18.7)
2	361 (20.1)	405 (21.0)	766 (20.6)
3	354 (19.7)	405 (21.0)	759 (20.4)
4	334 (18.6)	365 (18.9)	699 (18.8)
5	388 (21.6)	415 (21.5)	803 (21.6)
na	59	44	103

a Because of rounding, category percentages may not add to exactly 100.

cci = Charlson Comorbidity Index.

**TABLE II:** Frequency distribution a of patients and deaths by demographic characteristics

	*1996–1998*		*1999–2001*	
	*Patient [*n *(%)]*	*Deaths [*n *(%)]*	*Patient [*n *(%)]*	*Deaths [*n *(%)]*
Age (years)				
18–49	409 (22.1)	200 (48.9)	488 (24.7)	190 (38.9)
50–59	396 (21.4)	251 (63.4)	508 (25.8)	265 (52.2)
60–69	486 (26.2)	347 (71.4)	457 (23.2)	280 (61.3)
70+	561 (30.3)	446 (79.5)	520 (26.4)	377 (72.5)
cci				
0	577 (31.2)	208 (36.0)	680 (34.5)	212 (31.2)
1–5	347 (18.7)	209 (60.2)	361 (18.3)	166 (46.0)
6+	928 (50.1)	827 (89.1)	932 (47.2)	734 (78.8)
Income quintile				
1	356 (19.9)	249 (69.9)	339 (17.6)	196 (57.8)
2	361 (20.1)	233 (64.5)	405 (21.0)	231 (57.0)
3	354 (19.7)	240 (67.8)	405 (21.0)	234 (57.8)
4	334 (18.6)	218 (65.3)	365 (18.9)	195 (53.4)
5	388 (21.6)	263 (67.8)	415 (21.5)	220 (53.0)

a Because of rounding, category percentages may not add to exactly 100.

cci = Charlson Comorbidity Index.

**TABLE III:** Univariate analysis by death rate

*Covariate*	*Mortality rate (%)*	*Relative risk*	p *Value*
Period			
1996–1998	67.2	1.19	<0.0001
1999–2001	56.4	1.00	
Age (years)			
18–49	43.5	1.00	<0.0001
50–59	57.1	1.31	
60–69	66.5	1.53	
70+	76.1	1.75	
cci			
0	33.4	1.00	<0.0001
1–5	53.0	1.59	
≥6	83.9	2.51	
Income quintile			
1	64.0	1.00	0.2025
2	60.6	0.95	
3	63.6	0.99	
4	59.1	0.92	
5	60.1	0.94	
lhin^a^			0.5318

a Fourteen regions are not shown because of small numbers in some cases.

cci = Charlson Comorbidity Index; lhin = local health integrated network.

**TABLE IV:** Hazard ratio estimated by Cox regression model

*Covariate*	*Hazard ratio*	*95%cilower*	*95%ciupper*	p *Value*
Period				
1999–2001	0.90	0.83	0.98	0.0146
1996–1998	Ref			
Age (years)				
18–49	Ref			
50–59	1.39	1.22	1.59	<0.0001
60–69	1.75	1.54	1.99	<0.0001
70+	2.40	2.13	2.72	<0.0001
cci				
0	Ref			
1–5	0.79	0.71	0.89	<0.0001
≥6	1.94	1.71	2.19	<0.0001

Ref = reference variable.

**TABLE V:** Univariate analysis of duration of survival (in days)

		*Survival (days)*			
*Covariate*	*Patients (*n*)*	*Mean*	*sd*	*Median*	p *Value*
Period					
1996–1998	1852	1534	1244	1164	<0.0001
1999–2001	1973	1248	787	1354	
Age (years)					
18–49	897	1690	1016	1721	<0.0001
50–59	904	1521	984	1516	
60–69	943	1321	1037	1058	
70+	1081	1079	1031	693	
cci					
0	1257	1970	976	2000	<0.0001
1–5	708	1525	1096	1543	
≥6	1860	939	837	683	
Income quintile					
1	695	1301	1059	1078	0.0274
2	766	1406	1065	1287	
3	759	1336	1032	1136	
4	699	1458	1035	1435	
5	803	1419	1012	1385	
lhin					0.9303

sd = standard deviation; cci = Charlson Comorbidity Index; lhin = local health integrated network.
